# The impact of “early” versus “late” initiation of renal replacement therapy in critical care patients with acute kidney injury: a systematic review and evidence synthesis

**DOI:** 10.1186/s13054-016-1291-8

**Published:** 2016-05-06

**Authors:** Benjamin T. Wierstra, Sameer Kadri, Soha Alomar, Ximena Burbano, Glen W. Barrisford, Raymond L. C. Kao

**Affiliations:** Division of Internal Medicine, Department of Medicine, University of Calgary, Calgary, AB Canada; Harvard School of Public Health, Harvard University, Boston, MA USA; Division of Critical Care Medicine, Department of Medicine, Western University, 800 Commissioner’s Road East, London, ON N6A 5W9 Canada

**Keywords:** Meta-analysis, Intensive care units (ICUs), Acute kidney injury (AKI), Renal replacement therapy (RRT), Early, Late

## Abstract

**Background:**

The optimal timing of initiating renal replacement therapy (RRT) in critical illness complicated by acute kidney injury (AKI) is not clearly established. Trials completed on this topic have been marked by contradictory findings as well as quality and heterogeneity issues. Our goal was to perform a synthesis of the evidence regarding the impact of “early” versus “late” RRT in critically ill patients with AKI, focusing on the highest-quality research on this topic.

**Methods:**

A literature search using the PubMed and Embase databases was completed to identify studies involving critically ill adult patients with AKI who received hemodialysis according to “early” versus “late”/“standard” criteria. The highest-quality studies were selected for meta-analysis. The primary outcome of interest was mortality at 1 month (composite of 28- and 30-day mortality). Secondary outcomes evaluated included intensive care unit (ICU) and hospital length of stay (LOS).

**Results:**

Thirty-six studies (seven randomized controlled trials, ten prospective cohorts, and nineteen retrospective cohorts) were identified for detailed evaluation. Nine studies involving 1042 patients were considered to be of high quality and were included for quantitative analysis. No survival advantage was found with “early” RRT among high-quality studies with an OR of 0.665 (95 % CI 0.384–1.153, *p* = 0.146). Subgroup analysis by reason for ICU admission (surgical/medical) or definition of “early” (time/biochemical) showed no evidence of survival advantage. No significant differences were observed in ICU or hospital LOS among high-quality studies.

**Conclusions:**

Our conclusion based on this evidence synthesis is that “early” initiation of RRT in critical illness complicated by AKI does not improve patient survival or confer reductions in ICU or hospital LOS.

**Electronic supplementary material:**

The online version of this article (doi:10.1186/s13054-016-1291-8) contains supplementary material, which is available to authorized users.

## Background

Acute kidney injury (AKI) is a medical complication associated with significant morbidity and mortality in critically ill patients [1–3]. AKI is common in critical illness, and severe AKI is associated with up to 60 % hospital mortality [4]. Renal replacement therapy (RRT) within the intensive care unit (ICU) is conducted as either intermittent hemodialysis or continuous renal replacement therapy (CRRT). Traditional indications for RRT require the development of overt clinical manifestations of renal insufficiency, such as acidosis, electrolyte disturbances (most notably hyperkalemia), uremic complications (encephalopathy or pericarditis), and volume overload unresponsive to aggressive medical management. In spite of research and increasing clinical experience with dialysis, the optimal time to initiate RRT in the course of critical illness complicated by AKI is unclear.

The notion of “early” RRT is to initiate dialysis therapy before nitrogenous and other metabolic products accumulate to the degree where they become relatively resistant to therapy [5, 6]. Despite the intuitive rationale for “early” RRT, there is limited evidence to guide clinicians on the optimal time to initiate RRT in critical illness. Neither standard clinical parameters nor research into novel clinical biomarkers has emerged to clearly define an ideal time or clinical picture where the initiation of RRT optimizes patient outcomes. Earlier initiation of RRT must be balanced with potential patient harm associated with RRTs. Protocolled use of hemofiltration for 96 h in patients with septic shock admitted to an ICU regardless of their renal function suggests that “early” RRT can be associated with negative patient outcomes [7]. As a result, research into “early” RRT includes multiple definitions of *early* that reflect a potpourri of time factors, biochemical markers, and clinical parameters in an attempt to balance the risks of initiating RRT with the benefits expected from supporting renal function during critical illness.

The authors of two earlier meta-analyses pooled available data on this topic to suggest that “early” RRT improves survival in critical illness. Seabra et al. [8] identified 23 studies (5 randomized controlled trials [RCTs]/quasi-RCTs, 1 prospective study, and 17 retrospective cohort studies) and concluded that “early” initiation of RRT was associated with 28 % mortality risk reduction (relative risk [RR] 0.72, 95 % CI 0.64–0.82, *p* < 0.001). Karvellas et al. [9] identified 15 studies (2 RCTs, 4 prospective studies, and 9 retrospective cohort studies) and reached similar conclusions, reporting a significant improvement in 28-day mortality with “early” RRT (OR 0.45, 95 % CI 0.28–0.72, *p* < 0.001). However, the overall findings were not congruent with the subgroup analysis of randomized trials (RR 0.64, 95 % CI 0.4–1.05, *p* = 0.08), where there was a signal that “early” RRT was not associated with a significant survival advantage. This has diminished clinical confidence in the conclusions reached by the earlier meta-analyses, and consequently “early” RRT in critical illness remains a controversial therapeutic intervention.

Since 2012, additional studies have been published that do not support the conclusions of the previous meta-analyses, and this has further diminished the confidence in the previous conclusions that suggested a survival benefit in critical illness associated with “early” RRT. We conducted a systematic review and evidence synthesis to investigate whether “early” versus “late” initiation of RRT in critically ill patients with AKI improves patient survival and selected secondary outcomes for potential signals to suggest that “early” RRT may reduce patient morbidity or enhance illness recovery. Our goal was to identify the highest-quality studies on this topic and use a pooled meta-analysis of these studies to inform our conclusions.

## Methods

### Search strategy

This study was conducted in accordance with the Preferred Reporting Items for Systematic Reviews and Meta-Analyses (PRISMA) guidelines [10] (see Additional file 1: Figure S1 for PRISMA checklist). Our null hypothesis was that “early” initiation of RRT does not improve patient survival in critical care patients with AKI. This systematic review was not registered, and a protocol does not exist. The PubMed and Embase databases were searched to identify published articles following four broad themes: AKI, RRT, time of initiation, and critical illness (see Additional file 2: Table S1 for search terms). SK is a National Institutes of Health (NIH) physician and requested the NIH librarian to provide oversight for the search strategy. Our search was limited to English-language-only, full-text primary research publications (including abstracts with full text availability) reporting findings of clinical trials and observational studies (cohort and case-control design) published between January 1985 and November 2015. Studies before 1985 were not actively sought, owing to a low likelihood of relevance to modern RRTs and critical care practices.

### Study selection

References were screened and excluded if they were small case reports or observational studies (fewer than 10 subjects), were not focused on critically ill adult patients, did not report mortality data, involved basic science data, or did not clearly distinguish between “early” and “late” groups. This task was divided among the authors. A second evaluation led by the senior author (RLCK) was conducted to evaluate study quality. Studies were designated as being of “high quality” or “low quality.” Studies were assigned a “low-quality” rating if there was no illness severity assessment between cohorts or at the time of randomization (*n* = 8), significant differences (*p* < 0.05) between cohort groups (*n* = 7) at baseline, incomplete basic demographic data at baseline (*n* = 6) to exclude baseline differences, or a Newcastle-Ottawa Quality Assessment (NOQA) Scale [11] for cohort studies rating less than 7 (*n* = 6). The senior author (RLCK) was the arbiter in cases of disagreement. Only high-quality studies were included in quantitative meta-analysis of the primary and secondary outcomes.

### Primary and secondary outcomes

The primary outcome of interest was mortality at 1 month (pooling outcomes for mortality at 28 or 30 days, depending on what was reported by the primary authors). In addition to mortality, we analyzed selected secondary outcomes, including ICU length of stay (LOS) and hospital LOS. Secondary outcomes were not consistently reported for all studies, and only studies with applicable data were included in our pooled analysis. Weighted means were calculated as a product of the number of patients and mean duration to reach a total and represented as a total of patient-days per study. These values were summed and divided by the total number of patients from all included studies to reach weighted mean duration of LOS for both hospital and ICU LOS metrics. A similar process was used to derive the mean weighted illness severity scores. Other potentially relevant secondary outcomes, including mechanical ventilation requirements, vasopressor requirements, and renal recovery rates, were considered, but these variables were inconsistently reported and commonalities could not be reached among the heterogeneous parameters that were available.

### Definition of “early” versus “late”

*Early* was defined on the basis of criteria used by the original authors in their respective studies. We accepted a broad definition of *early* based on biochemical markers according to RIFLE classifications (risk, injury, failure, loss of function, and end-stage kidney disease), Acute Kidney Injury Network (AKIN) stages, or time-based cutoffs (e.g., within a defined time from ICU admission or development of a biochemical “start time”). Accepting a broad definition of *early* was intended to optimize the potential for identifying an effect associated with “early” RRT. A limitation of this approach is that “early” according to one study investigator might be considered “late” by another study investigator. “Late” RRT criteria involved either usual practice or expectant care (i.e., no RRT initiated). “Usual practice” generally involved implementing RRT following the development of classic RRT indications unresponsive to medical management.

### Statistical analysis

The quality of cohort trials was assessed using the NOQA Scale (range from 0 to 9, with 9 indicating the highest quality) [11]. The NOQA Scale for cohort studies assesses the domains of population selection, comparability of cohorts, and outcome assessment. A meta-analysis was conducted using the high-quality studies to calculate the pooled OR for mortality at 1 month. A random effects model was used because of the significant heterogeneity between studies on this topic. A random effects model is indicated when study populations differ in ways that could impact the results. Heterogeneity was assessed on the basis of the *Q* value and *I*^2^ and τ^2^ statistics. A *p* value less than 0.05 was considered statistically significant. All analyses were performed using Comprehensive Meta-Analysis version 3.3.070 software (www.meta-analysis.com; Biostat, Englewood, NJ, USA).

## Results

The systematic literature search yielded 2405 references that were subsequently refined to 36 studies eligible for inclusion in this meta-analysis (see Additional file 3: Figure S2 for article selection breakdown). These references included 7 RCTs [7, 12–17], 10 prospective cohort studies [18–27], and 19 retrospective cohort studies [28–46]. Only nine studies met our criteria for high quality [7, 12, 14–17, 21, 35, 40]. A summary of the fundamental characteristics of all evaluated studies is provided in Table 1.

**Table 1:** Trial Summary Table by Study Type (*n*=36)

Author, Year	Study Design	Country	Duration	Exclusion	Patient Population	Patients (n)			Age (mean) yrs	Illness Severity Score	Early RRT Criteria	Late RRT Criteria	Study Quality	Primary Outcome
						Total	Early RRT	Late RRT						
Randomized Trials														
Bouman, 2002 [12]	RCT, two-center study	Netherlands	May 1998 - Mar 2000	Pre-existing renal disease	Multisystem	106	70	36	EHV: 68; ELV: 70; LLV: 67	EHV: SOFA 10.3 - APACHE2=23.5, ELV: SOFA 10.1 - APACHE2=21.7; LLV: SOFA 10.6 - APACHE2=23.6	TIME: Early < 12 h (200ml); Early Low Vol < 12 h (100-150ml)	TIME: Late > 12h	HIGH	28 d mortality: EHV: 9/35(26%) died, ELV: 11/35(31%) died, LLV: 9/36(25%) died; *p*=0.8
Durmaz, 2003 [13]	RCT	Turkey	Sept 1999 - Aug 2001	Age<18, chronic dialysis	Post Cardiac Surgery	44	21	23	Early 58; Late 54	NR	BIOCHEM: Cr rise >10% from pre-op level within 48hrs of surgery	Cr rise >50% from pre-op level; or Urine output <400ml/24hrs with coexistent K+/H+ unresponsive to med mgmt	LOW	Hospital mortality: Early 1/21 (4.8%) died, Late 7/23 (30.4%) died *p*=0.048; Favors Early
Sugahara, 2004 [14]	RCT	Japan	Jan 1995 - Dec 1997	Pregnancy, Bili > 5mg/dL, Mental disorder, Cancer, Early recovery of urine output >30ml/kg/hr prior to RRT	Post Cardiac Surgery	28	14	14	Early: 65; Late: 64	Early: APACHE2=19; Late: APACHE2=18	BIOCHEM: UOP <30ml/hr × 3hrs OR UOP <750ml/day; Mean time to RRT start 18d±0.9 post op	UOP<20ml/hr × 2hrs+ OR UOP <500ml/day; Mean time to RRT start 1.7d±0.8 post op	HIGH	14 d mortality: Early 2/14 died (14%), Late 12/14 died (86%); *p*<0.01 Favors Early
Payen, 2009 [7]	RCT, multicenter	France	Jan 1997 - Jan 2000	Age<18, chronic dialysis, pregnant, moribund state, prior immunosuppressive therapy	Multisystem	76	37	39	Early 58 Late 59	Early: SOFA 11.6- SAPS2 54.3; Late: SOFA 10.4- SAPS2 52.4	TIME: Protocolized RRT × 96hrs w/ diagnosis of ‘sepsis’. Mean time to initiation of RRT not specified	Control = No RRT unless metabolic renal failure & classic indications for RRT present	HIGH	Early 20/37 (54%) died, Late 17/37 (44%) died; *p* = 0.49
Jamale, 2013 [15]	RCT, single center	India	April 2010 - July 2012	Required urgent dialysis at time of randomization	Multisystem	208	102	106	Early 43 Late 42	Early: SOFA 7.3; Late: SOFA 8.2	BIOCHEM: Cr > 618μmol/L	Classic indications for RRT, Symptomatic uremia unresponsive to med mgmt	HIGH	Mortality: Early 21/102 (20.5%) died, Late 13/106 (12%); *p*=0.2
Combes, 2015 [16]	RCT, multicenter	USA	2009-2012	<18, Pregnant, Chronic RRT, Weight >120kg, SAPS II>90 (i.e. moribund)	Post Cardiac Surgery	224	112	112	Early 61 Late 58	Early: SOFA 11.5- SAPS2=54; Late: SOFA 12.0- SAPS2=55.1	TIME: RRT initiated <24hrs and continued for min of 48hrs; Mean time to randomization 12hrs	Classic indications for RRT, Lifethreatening metabolic derangements unresponsive to med mgmt	HIGH	Mortality: Early 40/112 (36%) died, Late 40/112 (36%) died; *p* = 1.0
Wald, 2015 [17]	RCT, multicenter	Canada	May 2012 - Nov 2013	Intoxication requiring RRT, Limited resuscitation directives, RRT within the previous 2 months, RPGN, Obstructive uropathy, > 48hrs to doubling time of Cr	Multisystem	100	48	52	Early 62 Late 64	Early: SOFA 13.3 Late: SOFA 12.8	TIME: Time from randomization < 12h; Mean time to RRT = 9.7hrs	Intensivist judgement regarding hyperkalemia, volume overload, acidemia refractory to medical therapy, Uremic symptoms Mean time to RRT=32hrs	HIGH	Mortality: Early 16/48 (33%) died, Late 19/52 died; *p* = 0.74
RCT Totals						786	404	382						Pooled mortality: Early 120/404 (29.7%), Late 117/382 (30.6%); *n*=7
Prospective Trials														
Liu, 2006 [18]	Prospective Observational Multicentre	Multi countries	Feb 1999 - Aug 2001	GFR<30ml/min/1.73m2	Multisystem	243	122	121	Early 54 Late 58	NR	Azotemia defined by BUN<76mg/dL	Azotemia defined by BUN>76mg/dL	LOW NOQA=6	28 d mortality: Early 43/122(35%) died vs Late 50/121(41%) *P*=0.09 Favors Early
Iyem, 2009 [19]	Prospective Observational cohort	Turkey	May 2004 - April 2007	Preexisting renal disease and pre operative high levels of urea and creatinine	Post cardiac surgery	185	95	90	Early: 64; Late: 62	NR	TIME: Evidence of 50% increase in BUN, low urine output (<0.5mL/kg/h) triggering RRT started < 48hrs	TIME > 48hrs to start of RRT for similar markers of renal failure managed medically for minimum 48hrs	LOW NOQA=7	In hosp mortality: Early 5/95(5%) died, Late 6/90(7%) died; NS
Bagshaw, 2009 [20]	Prospective Observational Multicentre (BEST Kidney)	23 countries	Sept 2000 - Dec 2001	Pre existing chronic RRT, drug toxicity, age <12	Multisystem	1227	959	268	Early: 60, Delayed: 63, Late: 64; *p*=0.003	Early: SOFA 10.9- SAPS2=53.5 Delayed: SOFA 11.1- SAPS2=46 Late: SOFA 10.7- SAPS2=43.1; *p*=0.04	TIME: Early RRT started for azotemia (Urea>30mmol/L or low urine output × 12h) <2d (*n*=785), Delayed RRT started 2-5d (*n*=174) from ICU admission	RRT started >5d from ICU admission	LOW NOQA=7	Hosp mortality: Early 462/785(59%) died, Delayed 108/174(62%) died, Late 195/268(72%) died; *P*<0.0011 Favors Early
Shiao, 2009 [21]	Prospective Observational Multicentre	Taiwan	Jan 2002 - Dec2005	Prior dialysis, without surgery, or surgery did not involve abdominal cavity. History of renal trasplant	Major abdominal surgery	98	51	47	Early: 65; Late: 68	Early: SOFA 8.3- APACHE2=18.2; Late: SOFA 8.5- APACHE2=18.8	BIOCHEM: RIFLE criteria: RISK or pre-RISK criteria (Mean Time to RRT from ICU Admit = 7.3d)	RIFLE criteria: INJURY or FAILURE criteria (Mean Time to RRT from ICU Admit=8.4d)	HIGH NOQA=7	Hosp mortality: Early 22/51(43%), Late35/47(75%); *p*=0.0028 Favors Early
Sabater, 2009 [22]	Prospective Observational	Spain	2 years	NR	Multisystem	148	44	104	All patients mean = 60; NR	Early: APACHE2=26; Late: APACHE2=24	BIOCHEM: RRT initiated for RIFLE: RISK & INJURY; (Mean RRT start 2.2d post ICU admit)	RRT initiated for RIFLE: FAILURE; (Mean RRT start 6.4d post ICU admit)	LOW NOQA=7	Mortality: Early 21/44 died, Late 68/104 died. *P*=0.047 Favors Early
Elseviers, 2010 [23]	Prospective Observational Multicentre	Belgium	2001-2005	Pre existing renal disease (Cr<1.5mg/dl), reduced kidney size on ultrasound	Multisystem	1303	653	650	Early 64; Late 67	Early: SOFA 9.9- APACHE2=25.2; Late: SOFA 8.5- APACHE2=5.2, *p*=0.001	BIOCHEM: Unspecified SHARF scoring criteria w/serum Cr > 2mg/dL	Conservative approach = No RRT	LOW NOQA=5	Mortality: Early 379/653 (58%) died, Late 280/650 (43%) died; *p*<0.001 Favors Late
Vaara, 2012 [24]	Prospective Observational Multicentre (FINNAKI Study)	Finland	Sep 2011 - Feb 2012	NR	Sepsis, Cardiogenic Shock	261	NR	NR	NR	Survivors: SAPS2=47; Non-survivors: SAPS2=66	TIME: Time<24hrs from ICU admit	Time> 24hrs from ICU admit	LOW NOQA=5	OR for late 2.69 (1.07-6.73, *p*=0.035). Favors Early
Perez, 2012 [25]	Prospective Observational	Spain		NR	Sepsis	244	135	109	Early 62; Late 62	Early: SOFA 12; Late: SOFA 11	TIME: Time from ICU admission to RRT < 48h	TIME >48hrs	LOW NOQA=5	90 d mortality: Early 71/135(53%) died, Late 78/109(72%) died; *p*=0.003. Favors Early
Lim, 2014 [27]	Single Centre Prospective Cohort	Singapore	Dec 2010 - April 2013	Chronic dialysis patients, Dialysis initiated prior to ICU admission	Medical & Surgical patients	140	84	56	Early 60; Late 64	Early: SOFA 7; Late: SOFA 11; *p*=0.001	BIOCHEM: AKIN stage 1 or 2 AND compelling indication or AKIN stage 3 (Cr≥354μmol/l or Cr>300% baseline w/urine <0.3cc/kg/h for 24h or anuria >12h)	Traditional indications: K>6mmol/L, Urea ≥30mmol/L, pH<7.25, Bicarb <10mmol/L, Pulm edema, Uremic encephalopathy/pericarditis	LOW NOQA=6	Hosp mortality: Early 36/84(43%) died, Late 37/56(66%) died; *p*=0.007 Favors Early
Jun, 2014 [26]	Nested Observational, Multi-Centre Study ‘RENAL’ Study Group	NZ, Australia	Dec 2005 - Nov 2008	Age<18, Prior RRTduring admission, Prior RRT for CKD	Sepsis	439	219	220	Early 65; Late 64	Early: SOFA: 2.0- APACHE3=107, Late: SOFA 2.1- APACHE3=100, *P*<0.001	TIME: AKI diagnosis to randomization < 17.6 hrs	Time from AKI diagnosis to randomization >17.6hrs	LOW NOQA=6	28 d mortality: Early 82/219(37%) died; Late 84/220(38%) died (*p*=0.923) NS
PROSPECTIVE TOTALS						4288	2362	1665						Pooled mortality: Early 1229/2362 (52%), Late 833/1665 (50%); *n*=10
Retrospective Trials														
Gettings, 1999 [28]	Retrospective cohort	USA	1989 - 1997	CRRT duration <48hrs, Pediatric patients, Incomplete records	Trauma	100	41	59	Early 40; Late 48	Early ISS = 33.0; Late ISS = 37.2	BIOCHEM: BUN < 60mg/dL AND Oliguria, Vol overload, Electrolytes, Uremia; Mean RRT start post admission day 10; *p*<0.0001	BUN > 60 mg/dL AND Oliguria, Vol overload, Electrolytes, Uremia; Mean RRT start post admission day 19	LOW NOQA=5	Hosp mortality: Early 25/41(61%) died, Late 47/59(80%) died; *p*=0.041 Favors Early
Elahi, 2004 [29]	Retrospective cohort	UK	Jan 2002 - Jan 2003	Preexisting renal disease	Post cardiac surgery	64	36	28	Early 69; Late 68	NR	BIOCHEM: Low urine output = less than 100 ml within 8h after surgery;Mean RRT start 0.78 days	Traditional indications: Urea ≥30mmol/L, Cr Elahi, 2004 [29] ≥250mmol/L, K > 6.0mEq/L; Mean RRT start 2.5 days	LOW NOQA=6	28 d mortality: Early-8/36 died (22%), Late-12/28 (43%); *p*<0.05 Favors Early
Demirkilic, 2004 [30]	Retrospective cohort	Turkey	Mar 1992 - Sep 2001	NR	Post Cardiac Surgery	61	34	27	NR p=0.3	NR	BIOCHEM: Low urine output = less than 100ml within 8hrs post op; Mean RRT start 0.88 days	Cr≥5mg/dL, or K>5.5 mEq/L w/med mgmt; Mar 92-Jun 96; Mean RRT start 2.56 days	LOW NOQA=6	Hosp mortality: Early 8/34(23%), Late 15/27(56%); *P*=0.016 Favors Early
Wu, 2007 [32]	Retrospective cohort	Taiwan	July 2002- Jan2005	Hepatorenal syndrome from cirrhosis, liver trasplant, cardiopolmunary resuccitation	Acute liver failure	80	54	26	Early 55; Late 63; p=0.03	Early: SOFA 12.4- APACHE2=18.2; Late: SOFA 13.2- APACHE2=20.5	BIOCHEM: BUN < 80 mg/dL AND traditional indications present	Traditional indications present with BUN > 80mg/dL	LOW NOQA=6	30 d mortality: Early 34/54(63%) died vs Late 22/26(85%) died; *P*=0.04 Favors Early
Andrade, 2007 [31]	Retrospective cohort	Brazil	2002-2005	Patients who did not have both AKI and respiratory failure believed secondary to leptospirosis	Leptospirosis	33	18	15	Early 42; Late 44	Early: APACHE2=24.5; Late: APACHE2=26	TIME: Mean time to RRT = 265 min	Mean time to RRT = 1638 min	LOW NOQA=5	Hosp mortality: Early 3/18(17%) died, Late 10/15(67%) died; *P*=0.01 Favors Early
Manche, 2008 [33]	Retrospective cohort	Malta	1995-2006	NR	Post Cardiac Surgery	71	56	15	Early 66; Late 63	NR	BIOCHEM: Urine output<0.5ml/kg/hr unresponsive to med mgmt; Mean RRT start 8.6hrs post-op	Oliguria (output < 0.5ml/Kg/hr) refractory to med mgmt; Mean RRT start 41.2hrs post-op	LOW NOQA=6	Mortality: Early 14/56(25%) died, Late 13/15(87%) died; *P*=0.0000125 Favors Early
Lundy, 2009 [34]	Retrospective cohort	US	Nov 2005 - Aug 2007	Preexisting renal disease, burn size of less than 40% Non-thermal injury, lithium toxicity	Severe Burned patients	57	29	28	Early 27; Late 38 P=0.06	Early: SOFA 13- APACHE2=35; Late: SOFA 13- APACHE2=36	BIOCHEM: AKIN stage 2(+shock)/3; Mean time from admit to RRT = 17 days	Mean time from admit to AKIN stage 2(+shock)/3 but not dialyzed = 23 days	LOW NOQA=6	28 d mortality: Early 9/29(31%) died, Late 24/28(85%) died; *P*<0.002; Favors Early
Carl, 2010 [35]	Retrospective cohort	US	2000-2004	Baseline eGF0R <30ml/min, Age <18 & prisoners	Sepsis	147	85	62	Early 52; Late 56	Early: APACHE2=24.8; Late: APACHE2=24.7	BIOCHEM: BUN <100mg/dL + AKIN stage >2; Mean ICU stay prior to RRT =6.3days	BUN > 100mg/dL + AKIN stage >2; Mean ICU stay prior to RRT=12.3days	HIGH NOQA=7	28 d mortality: Early 44/85(52%) died, Late 42/62(68%); *P*<0.05 Favors Early
Chou, 2011 [37]	Retrospective cohort ‘NSARF’ database	Taiwan	Jan 2002 - Oct 2009	Age< 18, ICU stay <2days, RRT < 2days	Sepsis + AKI	370	192	178	Early 64; Late 66	Early: SOFA 10.8- APACHE2=12.3; Late: SOFA 11.6- APACHE2=14.0	BIOCHEM: RIFLE criteria: RISK or pre-RISK criteria	RIFLE criteria: INJURY or FAILURE criteria	LOW NOQA=6	Hosp mortality: Early 135/192(71%) died, Late 124/178 (70%) died (*P*=0.98)
Vats, 2011 [38]	Retrospective cohort	USA	Jan1999 - Feb 2006	Renal transplant, Pre-morbid ESRD on dialysis, RRT<24h, insufficient data	Multisystem	230	NR	NR	All patients mean = 66 NR	NR	TIME: Time from AKI to RRT < 6 days	Time from AKI to RRT≥6d	LOW NOQA=5	OR for Late Mortality (>6d) 11.66 (1.26-107.9) *P*=0.0305, Favors Early
Ji, 2011 [36]	Retrospective cohort	China	Ap 2004 - Mar 2009	Patients readmitted post discharge, Discharged against medical advice, Death <24hrs	Post cardiac surgery	58	34	24	Early 64; Late 62	Early: APACHE3=69.3; Late: APACHE3=88.2 *p*<0.001	TIME: Time from urine output <0.5ml/kg/h to RRT<12h; Mean oliguria to start of RRT 8.4hrs	Urine output <0.5ml/kg/h & Time to RRT>12h post oliguria; Mean oliguria to start of RRT 21.5hrs	LOW NOQA=6	Hosp mortality: Early 3/34 (9%) died, Late 9/24 (37%); *p*=0.02 Favors Early
Shiao, 2012 [41]	Retrospective cohort ‘NSARF’ database	Taiwan	Jan 2002 - Apr 2009	Dialysis before surgery, ESRD	Surgical	648	436	212	Early 62; Late 66; P=0.009	Early: SOFA 11.4- APACHE2=12.7; Late: SOFA 11.3- APACHE2=12.8	TIME: Time to development of tradtional RRT indications < 3d; Mean time to start of RRT 1.4days	Traditional RRT indications AND start of RRT > 3 days; Mean time to start of RRT 18days	LOW NOQA=6	Hosp mortality: Early 236/436 (54%) died, Late 143/212 (67%) died; *P*=0.001 Favors Early
Chon, 2012 [40]	Retrospective cohort	South Korea	Apr 2009 - Oct 2010	Liver cirrhosis, Pre existing chronic	Sepsis	55	36	19	Early 63; Late 62	Early: SOFA 13.5- APACHE2= 28.7; Late: SOFA 12- APACHE2=28.3	TIME: Time to RIFLE ‘Injury’/‘Failure’ < 24hrs; Mean time to RRT=12.5hrs	Time to RIFLE ‘Injury’/‘Failure’ > 24hrs; Mean time to RRT= 42.2hrs	HIGH NOQA=7	28 d mortality: Early 7/36(38%), Late 9/19(47%); P=0.03 Favors Early
Boussekey, 2012 [39]	Retrospective cohort	France	Jan 2008 - Dec 2010	Early trasfer to another unit	Multisystem	110	67	43	Early 62; Late 66	Early: SOFA: 11.1- SAPS2=70; Late: SOFA 8.8- SAPS2=57; *p*=0.002	TIME: Time from RIFLE- ‘Injury’ to RRT < 16hrs; Mean time to RRT=6hrs	Time from RIFLE-‘Injury’ to RRT > 16hrs; Mean time to RRT=64hrs	LOW NOQA=7	28 d mortality: Early-28/67 (41%), Late- 28/43 (65%); *P *= 0.0425 Favors Early
Suzuki, 2013 [43]	Retrospective cohort	Japan	Jan 2009 - Feb 2013	<18, RRT for ESRD	Sepsis, Cardiogenic Shock	189	52	137	All patients mean = 72 NR	All patients SAPS II Mean= 57	BIOCHEM: RIFLE ‘Risk’	RIFLE ‘Injury’ or ‘Failure’	LOW NOQA=6	Early: OR 0.361 (95 % CI 0.17–0.78); *P* = 0.009, Favors Early
Shum, 2013 [43]	Retrospective cohort	China	Jan 2008 - Jun 2011	Age<18, Chronic dialysis, RRT prior to ICU	Sepsis	120	31	89	qEarly 74; Late 73	Early: SOFA 12- APACHE4=119; Late: SOFA 13- APACHE4=133; *P*=0.011	BIOCHEM: sRIFLE-‘pre- Risk’ or ‘Risk’ criteria; Mean time from ICU admit to RRT =20.7hrs, *P*=0.056	sRIFLE ‘Injury’ or ‘Failure’ criteria; Mean time from ICU admit to RRT=10.8hrs	LOW NOQA=6	28 d mortality: Early-15/31 died (48.4%), Late- 43/89 died (48.3%); *P*=0.994
Tian, 2014 [46]	Retrospective cohort	China	Nov 2009 - Dec 2011	Age < 12, Chronic renal disease, Terminal illness,0 Pre-admit CRRT, ICU stay < 72hrs	Sepsis - AKIN 1	49	23	26	Early 48; Control 54	Early: SOFA 7.6- APACHE2=12.9; Control: SOFA 8.4- APACHE2=15.3	BIOCHEM: AKIN 1 (Cr≥26.4μmol/L or >150- 200% baseline & urine <0.5cc/kg/h for >6h)	No RRT (Control): Patients refused CRRT for “personal reasons”	LOW NOQA=6	28 d mortality: Early 5/23(22%) died, Control 11/26 (42%) died (NS)
					Sepsis - AKIN 2	52	31	21	Early 54; Control 61	Early: SOFA 9.3- APACHE2=19; Control SOFA 9.6- APACHE2=18.3	AKIN 2 (Cr>200-300% baseline & urine <0.5cc/kg/h for >12h)	No RRT (Control): Patients refused CRRT for “personal reasons”		28 d mortality: Early 12/31 (39%) died, Control 14/21 (67%) died; *P*<0.05 Favors Early
					Sepsis - AKIN 3	59	46	13	Early 50; Control 55	Early SOFA 10- APACHE2=21.8; Control SOFA 11.2- APACHE2=20.5	AKIN 3 (Cr≥354μmol/L or Cr>300% baseline w/urine <0.3cc/kg/h for 24h or anuria >12h)	No RRT (Control): Patients refused CRRT for “personal reasons”		28 d mortality: Early 31/46(67%) died, Control 11/13(85%) died; NS
Serpytis, 2014 [45]	Retrospective cohort	Lithuania	2007-2011	NR	Sepsis	85	42	43	All patients mean = 72 NR	NR	TIME: Time from anuria to RRT < 12hrs	Time from anuria to RRT > 12hrs	LOW NOQA=5	Mortality: Early 30/42 (71%) died, Late 39/43(91%) died; *p*=0.028; Favors Early
Gaudry, 2014 [44]	Retrospective cohort	France	Jan 2004 - Nov 2011	Age<18, limitation in medical therapy, death<24hrs, chronic renal insufficiency, RRT prior to ICU, kidney transplant, lithium toxicity, multiple myeloma	Sepsis	203	91	112	Early 65; Late 65	Early: SOFA 9- SAPS2=60; Control SOFA 8- SAPS2=55, *P*<0.01	BIOCHEM: RRT criteria: Cr≥300μmol/L, Urea>25mmol/L, K>6.5mmol/L, pH<7.2, Oliguria, Vol overload,	No RRT initiated/Criteria not met for RRT	LOW NOQA=5	Hosp Mortality: Early 44/91(48%) died, Control (No RRT) 29/112 (26%) died; *P*<0.001 Favors no RRT
Retrospective TOTALS						2841	1434	1177						Pooled mortality: Early 714/1434 (50%), Late 732/1177 (62.2%); *n*=19

*LEGEND*: *AKI* Acute kidney injury, *AKIN* Acute Kidney Injury Network, *APACHE* Acute Physiology and Chronic Health Evaluation, *Cr* Creatinine, *CRF* Chronic renal failure, *CRRT* Chronic renal replacement therapy, *eGFR* Estimated glomerular filtration rate, *EHV* Early High Volume, *ELV* Early Low Volume, *ESRD* End stage renal disease, *ICU* Intensive Care Unit, *LLV* Late Low Volume, *NOQA* Newcastle-Ottawa quality assessment, *NR* Not reported, *NSARF* National Taiwan University Hospital-Surgical ICU- Acute Renal Failure database, *RIFLE* Risk, Injury, Failure, Loss and End-stage, *RPGN* Rapidly progressive glomerularnephritis, *SAPS2* Squential Acute physiology Score, *SHARF* Stuivenberg Hospital Acute Renal Failure Score, *SOFA* Sequential Organ Failure Assessment, *UOP* Urine output

### Primary outcome

The observed pooled crude mortality rates varied significantly between the high- and low-quality studies. Among the high-quality studies, the pooled “early” RRT study group mortality rate was 34.6 % (192 of 555) compared with 40.2 % (196 of 487) in the pooled “late” RRT group. The low-quality studies demonstrated a pooled “early” RRT group mortality rate of 51.3 % (1871 of 3645) compared with 54.3 % (1486 of 2737) in the “late” RRT groups. The most frequently reported measurement of illness severity in the studies we analyzed was the Sequential Organ Failure Assessment (SOFA) score. The SOFA score has been correlated with critical care patient outcomes [47, 48], but it is not as robust as other scoring systems validated in predicting survival (e.g., Acute Physiology and Chronic Health Evaluation II [APACHE2] or Simplified Acute Physiology Score II [SAPS2]) [49]. The mean weighted SOFA scores in the high-quality studies were 10.2 and 10.4 in the “early” and “late” groups, respectively. SOFA scores were reported for 78 % of patients in the high-quality studies. Among the high-quality studies, the SOFA score appeared to correspond with an APACHE2 score of approximately 20 or a SAPS2 score of approximately 53 when these additional illness severity metrics were reported by the principal investigators. Unfortunately, more detailed quantitative evaluation of illness severity using APACHE2 or SAPS2 scores was not possible, owing to heterogeneous reporting methods between investigators and a lack of sufficient data. SOFA scores were reported for 65 % of the patients in the studies assigned low-quality ratings. The mean weighted SOFA scores in the “early” and “late” groups among the low-quality studies were comparable to those for the high-quality studies at 10.0 and 9.2, respectively. No further comments can be made regarding illness severity scores among the low-quality studies, owing to lack of homogeneous and sufficient data. Illness severity scores for all studies are summarized in Table 1.

**Fig. 1:**
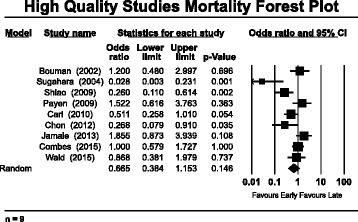
Mortality forest plot of pooled analysis of high-quality studies (*n* = 9)

**Fig. 2:**
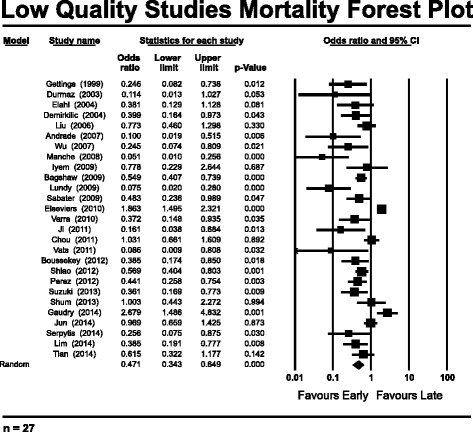
Mortality forest plot pooled analysis of low-quality studies (*n* = 27)

Pooled analysis of the high-quality studies (*n* = 9) indicates no mortality benefit with “early” versus “late” RRT, with an OR of 0.665 (95 % CI 0.384–1.153, *p* = 0.146) (Fig. 1). The bulk of the data in support of “early” RRT rests in the pooled low-quality studies (*n* = 27), with an OR of 0.471 (95 % CI 0.343–0.649, *p* < 0.001) (Fig. 2). Similarly to authors of previous meta-analyses, we found very high heterogeneity among studies on this topic. Heterogeneity was highest among the low-quality studies, reflected by a *Q* value of 163.8, *I*^2^ value of 84 %, and τ^2^ = 0.495 (*p* < 0.001). Among the high-quality studies, there continued to be statistically significant heterogeneity, with a *Q* value of 29.1, *I*^2^ value of 72.5 %, and τ^2^ = 0.481 (*p* < 0.001). Subgroup analysis of the high-quality studies according to ICU admission type and surgical [14, 16, 21] versus mixed medical admissions [7, 12, 15, 17, 35, 40] demonstrated no significant subgroup mortality benefits associated with “early” RRT (see Additional file 4: Figure S3a and b for forest plots by ICU admission type). Subgroup analysis among the high-quality studies was also conducted using the definition of *early* according to time criteria (hours or days) versus biochemical parameters (i.e., rising creatinine, uremia, oliguria) (see Additional file 5: Figure S4a and b for forest plots by biochemical or time definition of *early*). There were no significant effects observed in pooled mortality trends in studies that defined *early* by time criteria rather than on the basis of biochemical parameters.

### Secondary outcomes

**Fig. 3:**
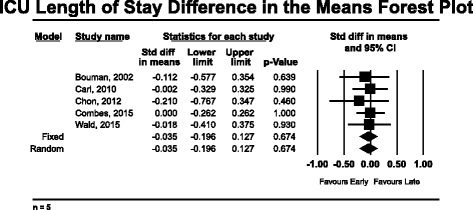
Forest plot of pooled analysis of standard difference of the means for intensive care unit length of stay (*n* = 5)

**Fig. 4:**
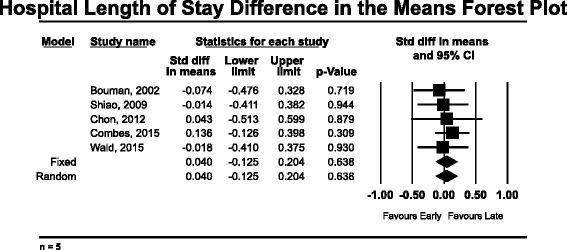
Forest plot of pooled analysis of standard difference of the means for hospital length of stay (*n* = 5)

The secondary outcomes analyzed included ICU LOS and hospital LOS. Five of the nine high-quality studies reported ICU LOS data [12, 16, 17, 35, 40]. The mean weighted ICU LOS in the “early” group was 9.4 days (*n* = 351), compared with 10.8 days (*n* = 281) in the “late” group. None of the studies reported a significant finding with respect to ICU LOS and “early” RRT. Pooled analysis for ICU LOS also demonstrated no significant change in ICU LOS associated with “early” RRT, with a standard difference in the means of −0.035 (95 % CI −0.196 to 0.127, *p* = 0.674) using a fixed effects model (*Q* = 0.598, *p* = 0.963) (Fig. 3). Hospital LOS was reported in five of nine high-quality studies [12, 16, 17, 21, 40]. The mean weighted hospital LOS in the “early” group was 19.3 days (*n* = 317), compared with 17.1 days (*n* = 266) in the “late” group. The pooled hospital LOS data do not reveal any significant difference in hospital LOS using a fixed effects model with a standard difference in the means of 0.040 (95 % CI −0.125 to 0.204, *p* = 0.638) (Fig. 4).

## Discussion

Despite several studies having been conducted on this topic over the last 30 years, a clear answer regarding the optimal timing of RRT in critical illness remains elusive. Our analysis does not confirm the conclusions of previous meta-analyses on this topic. Four studies [12, 14, 21, 35] in the high-quality group were previously included in the meta-analysis by Karvellas et al. [9], and only one study [12] was included in the meta-analysis by Seabra et al*.* [8]. The addition of four recently published studies [15–17, 40] and one high-quality study that was not previously included in meta-analysis [7] accounts for our results that differ from those of earlier authors. Our conclusions build on the concerns raised by both earlier meta-analyses that the results of cohort trials in favor of “early” RRT were not reproduced in methodologically more rigorous study designs (i.e., RCTs). In our further analysis we did not identify critical illness patient subgroups for whom “early” RRT might be more beneficial. Similarly, how one defines *early* (according to time or on the basis of biochemical characteristics) does not identify a survival advantage associated with “early” RRT compared with usual care. The optimal timing for initiation of RRT is not clarified on the basis of research evaluated to date.

The strength of our present analysis rests on our extensive literature search and strict classification according to study quality to limit risk of type I hypothesis testing error. Prior meta-analyses relied heavily on retrospective cohort study data that possessed incomplete preintervention data or preexisting significant differences in groups which predisposed the investigators to identify a survival difference attributed to “early” RRT that may have been accounted for by the preintervention population differences. We identified differences in the crude mortality rates between the high- and low-quality studies that are incompletely explained. The crude mortality rate differences may be explained by factors that are not adequately controlled for between the groups before the intervention of “early” versus “late” RRT (e.g., unreported regional institutional differences, variation in intensive care resources, institutional setting variability [academic versus community], or natural history variability of the diseases precipitating critical illness). In cohort trials, a difference in preintervention study groups indicates a critical methodological flaw that precludes deriving conclusions from their results. This is referred to as a type I error in hypothesis testing and may falsely attribute differences in outcomes to the study variable rather than the differences between cohorts that existed before analysis. Among high-quality studies, there was no survival advantage to “early” RRT with an OR of 0.665 (*p* = 0.146). Any inclusion of the low-quality study data would significantly pull the conclusion in favor of “early” RRT, which would represent fulfillment of a type I statistical hypothesis error. The strength of our work is that we vigorously guarded against this possibility.

Subgroup analysis of the high-quality studies did not reveal a survival benefit associated with either a surgical or medical critical care patient population. This conclusion remained the same regardless of whether *early* was defined by time or on the basis of biochemical parameters. Our secondary outcome analysis was limited by inconsistent and incomplete data reported across studies. Limited pooled analysis of the available data suggested that there was no significant effect on either ICU or hospital LOS associated with “early” RRT. Incomplete data does not permit us to evaluate additional secondary outcomes of interest (such as requirement for mechanical ventilation or rates of renal recovery) that might also be clinically relevant considerations factored into the decision to initiate RRT in critical illness.

By limiting our analysis to studies meeting high-quality criteria, we dismissed a large volume of research on this topic. A critique of our work is that we discarded studies for methodological shortcomings that others may feel should have been included. Most studies (*n* = 21) in the low-quality group were excluded for incomplete cohort data or significant preintervention differences between cohort groups. The decision to exclude these trials is less controversial than our decision to exclude cohort trials for an NOQA Scale rating less than 7 (*n* = 6). This is potentially controversial because the NOQA Scale has received criticism regarding its validity and applicability in meta-analysis cohort trial quality assessment [50]. The NOQA Scale has received positive endorsement from some authors [11], but detailed psychometric properties have not been published in peer-reviewed journals to date. Furthermore, our selection of an NOQA Scale rating less than 7 to identify low quality is arbitrary. Our rationale for selecting this cutoff was that it necessitates that at least one of the three NOQA Scale domains be seriously compromised, and we felt that this represented a significant bias predisposing the study results to committing a type I error pattern. Seabra et al. [8] attempted to assign a quality score to trials (0 = lowest quality to 5 = best quality) to evaluate this domain, but their methodology for score assignment was obscure and was not able to be replicated or directly compared with our methods. In qualitative comparison, the study assigned their top score [12] was included in our quantitative analysis; however, their second highest quality study [13] was excluded due to lack of reported illness severity scores between groups. Including studies with methodological errors does not advance scientific understanding of this topic and has contributed to the discordant findings on it.

Early studies on this topic were small and may have overestimated an effect size associated with “early” RRT based on the small size of the study populations. An example of this problem is the Sugahara et al. study [14], where 14-day mortality within the “early” group was 14 % (2 of 14), compared with 86 % (12 of 14) in the “late” group (*p* < 0.01). While this study was included in our quantitative analysis, the magnitude of the mortality benefit reported in this trial associated with “early” RRT has not been reproduced by subsequent investigators, for reasons that are not clear. In our review of the ongoing trials on this topic registered with the NIH (www.clinicalTrials.gov), we identified three trials [51–53] that may add to knowledge in this area. The methodology of all three active RCTs is roughly similar, with patients randomized from a point in time triggered by the development of biochemical renal injury reflected by a RIFLE grade of “failure” (at least one of rise in creatinine by minimum of 300 %, oliguria less than 0.3 ml/kg/h for 12 h, or anuria lasting more than 12 h). From this biochemical entry point, patients will be randomized to immediate initiation of RRT (goal time to RRT less than 12 h) or standard care (RRT initiated after failure of medical management to temporize metabolic derangements or volume overload). These study designs are similar to the design used by Wald et al. [17], included in our analysis, that was able to separate an “early” group to mean time to RRT of 9.2 h and a “late” RRT group with a mean time to RRT of 32 h after biochemical inclusion criteria were met. Wald et al. [17] did not identify a significant difference in mortality rates between their two groups (*p* = 0.74). These studies in process will add to the quantity of patients evaluated in this manner and will build on the availability of high-quality data on this topic. By clearly defining routine biochemical criteria associated with acute renal injury, they provide a practical method of renal injury assessment that can be determined by intensivists and nephrologists considering RRT.

## Conclusions

The results of our meta-analysis contradict the findings reported by previous authors [8, 9], and we conclude that “early” initiation of RRT in critically ill patients with AKI does not improve survival. This conclusion is derived from the pooled high-quality trial data and excludes data from cohort trials where there were methodological shortcomings that predisposed them to find an effect misattributed to the intervention. Pooled analysis of secondary outcomes did not demonstrate a statistical reduction in ICU or hospital LOS. Additional well-designed RCTs will provide greater confidence in these conclusions as optimal patient care practices progress in critical care. Clinical triggers for the initiation of RRT to optimize patient outcomes have not been clearly identified by current research. Meanwhile, intensivists and nephrologists are encouraged to refrain from lowering their clinical thresholds for implementing RRT in critical care patients with acute renal injury.

## Key messages

- High-quality trial data do not demonstrate improved survival using an “early” RRT approach in critical illness complicated by AKI.
- Lower-quality trial data demonstrate significantly higher mortality rates and form the basis for the bulk of support for “early” AKI.
- The optimal time to initiate RRT in critical illness remains undefined.
- A conservative approach to initiating RRT in critical illness is supported.

## Additional files

Additional file 1: Figure S1. Preferred Reporting Items for Systematic Reviews and Meta-Analyses (PRISMA) checklist. (PDF 51 kb) Additional file 2: Table S1. Search terms used during literature review. (DOCX 15 kb) Additional file 3: Figure S2. Article selection process. (PDF 86 kb) Additional file 4: Figure S3.
**a** Mortality forest plot of subgroup analysis of high-quality studies according to post-surgical ICU admission type (*n* = 3). **b** Mortality forest plot of subgroup analysis of high-quality studies according to medical ICU admission type (*n* = 6). (ZIP 120 kb) Additional file 5: Figure S4.
**a** Mortality forest plot of subgroup analysis of high-quality studies based on the definition of “early” according to time criteria (hours or days) (*n* = 4). **b** Mortality forest plot of subgroup analysis of high-quality studies based on the definition of “early” according to biochemical parameters (i.e., rising creatinine, uremia, oliguria) (*n* = 5). (ZIP 121 kb)

## Abbreviations

AKI: acute kidney injury
AKIN: Acute Kidney Injury Network
AMA: against medical advice
APACHE2: Acute Physiology and Chronic Health Evaluation II
ARDS: acute respiratory distress syndrome
BUN: blood urea nitrogen
CKD: chronic kidney disease
Cr: creatinine
CRF: chronic renal failure
CRRT: continuous renal replacement therapy
EHV: early high volume hemofiltration
ELV: early low volume hemofiltration
eGFR: estimated glomerular filtration rate
ESRD: end-stage renal disease
ICU: intensive care unit
ISS: illness severity score
LLV: late low volume hemofiltration
LOS: length of stay
NIH: National Institutes of Health
NOQA: Newcastle-Ottawa Quality Assessment
NR: not reported
NS: not significant
OR: odds ratio
PRISMA: Preferred Reporting Items for Systematic Reviews and Meta-Analyses
RCT: randomized controlled trial
RIFLE: risk, injury, failure, loss of function, and end-stage kidney disease
RPGN: rapidely progressive glomerulonephritis
RR: relative risk
RRT: renal replacement therapy
SAPS2: Simplified Acute Physiology Score II
SHARF: Stuivenberg Hospital Acute Renal Failure score below
SOFA: Sequential Organ Failure Assessment
sRIFLE: simple criteria for risk injury failure loss of function and end-stage kidney disease
UOP: urine output
NR: not reported
