# Supplementary material for: The impact of “early” versus “late” initiation of renal replacement therapy in critical care patients with acute kidney injury: a systematic review and evidence synthesis
Source: Crit Care. 2016 May 6;20:122. doi: 10.1186/s13054-016-1291-8 (PMC4858821; doi:10.1186/s13054-016-1291-8)
Supplement: Additional file 2: Table S1. — Search terms used during literature review. (DOCX 15 kb) [file 13054_2016_1291_MOESM2_ESM.docx]

Supplementary Index

Table 1. Search strategy terms and results

| PUBMED, searched 27 Nov 2015 | | |
| --- | --- | --- |
| 1 | Acute kidney injury [MeSH majr] OR “acute kidney”[ti] OR “acute renal”[ti] | 30,251 |
| 2 | Renal replacement therapy [MeSH majr] OR dialysis [ti] OR dialyzed [ti] OR dialyzing [ti] OR hemodialysis [ti] OR hemofiltration [ti] | 150,665 |
| 3 | Time to treatment [MeSH] OR Time factors [MeSH] OR Early [ti/ab] OR earlier [ti/ab] OR time [ti/ab] OR timing [ti/ab] OR accelerate [ti/ab] OR accelerated [ti/ab] OR accelerating [ti/ab] OR acceleration [ti/ab] OR late [ti/ab] | 3,659,624 |
| 4 | Combine #1 AND # 2 AND # 3 | 1,124 |
| 5 | Filters: English | 1,004 |
| 6 | Removal of duplicates (1) | 1,003 |

| EMBASE, searched 27 Nov 2015 | | |
| --- | --- | --- |
| 1 | Acute kidney failure OR Acute kidney tubule necrosis OR acute kidney OR acute renal | 69,225 |
| 2 | Renal replacement therapy OR dialysis OR dialyzed OR dialyzing OR hemodialysis OR hemofiltration | 221,772 |
| 3 | Time to treatment OR Time OR Early intervention OR Early OR Earlier OR Timing OR Accelerated OR Accelerating OR Acceleration OR Late | 5,026,658 |
| 4 | Critical Care OR Intensive Care Unit OR ICU | 306,234 |
| 5 | Combine #1 AND # 2 AND # 3 AND #2 | 1,897 |
| 6 | Limit to: English | 1,780 |
| 7 | Removal of duplicates (378) | 1,402 |
