# Supplementary figures and images for: The impact of “early” versus “late” initiation of renal replacement therapy in critical care patients with acute kidney injury: a systematic review and evidence synthesis
Source: Crit Care. 2016 May 6;20:122. doi: 10.1186/s13054-016-1291-8 (PMC4858821; doi:10.1186/s13054-016-1291-8)

## Supplementary Index

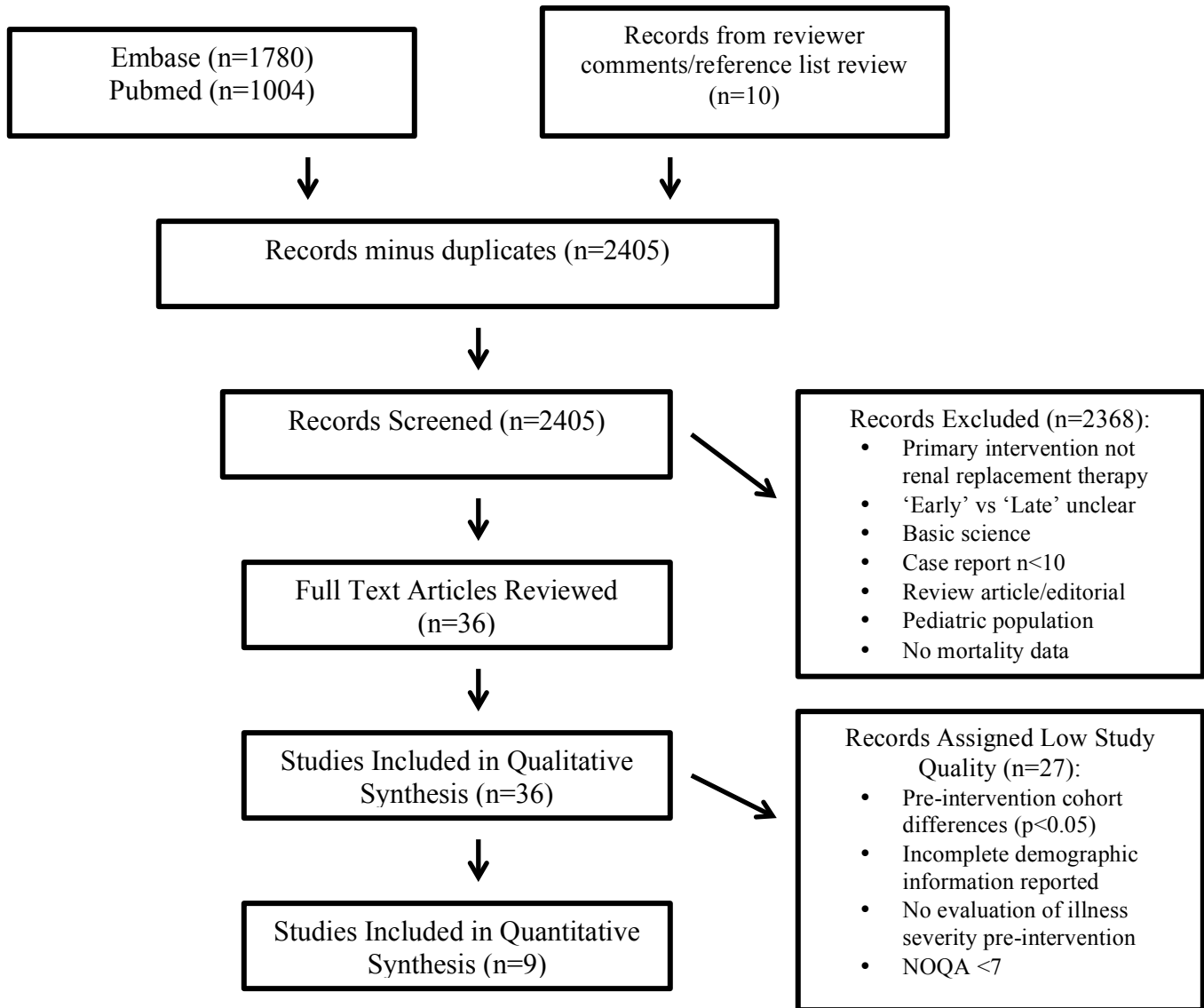

Figure 2. Search results article selection methodology

Supplement: Additional file 3: Figure S2. — Article selection process. (PDF 86 kb) [file 13054_2016_1291_MOESM3_ESM.pdf]
