# Supplementary material for: The impact of “early” versus “late” initiation of renal replacement therapy in critical care patients with acute kidney injury: a systematic review and evidence synthesis
Source: Crit Care. 2016 May 6;20:122. doi: 10.1186/s13054-016-1291-8 (PMC4858821; doi:10.1186/s13054-016-1291-8)
Supplement: Additional file 4: Figure S3. — a Mortality forest plot of subgroup analysis of high-quality studies according to post-surgical ICU admission type (n = 3). b Mortality forest plot of subgroup analysis of high-quality studies according to medical ICU admission type (n = 6). (ZIP 120 kb) [file 13054_2016_1291_MOESM4_ESM.zip › Supplementary Figure 3 a and b/Supplementary Index_Figure 3a_Surgical Subgroup Mortality Forest Plot.pdf]

Surgical Studies Subgroup Mortality Forest Plot

Model	Study name	Statistics for each study			
		Odds ratio	Lower limit	Upper limit	p-Value
	Sugahara (2004)	0.028	0.003	0.231	0.001
	Shiao (2009)	0.260	0.110	0.614	0.002
	Combes (2015)	1.000	0.579	1.727	1.000
Random		0.262	0.056	1.236	0.091

Odds ratio and 95% CI

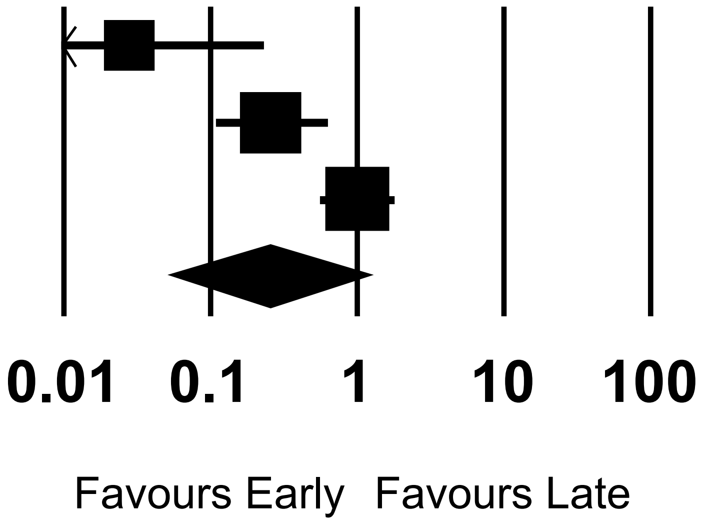

n = 3

Figure 3a. Surgical Subgroup Mortality Forest Plot Using Random Effects Model
