# Supplementary material for: The impact of “early” versus “late” initiation of renal replacement therapy in critical care patients with acute kidney injury: a systematic review and evidence synthesis
Source: Crit Care. 2016 May 6;20:122. doi: 10.1186/s13054-016-1291-8 (PMC4858821; doi:10.1186/s13054-016-1291-8)
Supplement: Additional file 4: Figure S3. — a Mortality forest plot of subgroup analysis of high-quality studies according to post-surgical ICU admission type (n = 3). b Mortality forest plot of subgroup analysis of high-quality studies according to medical ICU admission type (n = 6). (ZIP 120 kb) [file 13054_2016_1291_MOESM4_ESM.zip › Supplementary Figure 3 a and b/Supplementary Index_Figure 3b_Medical Subgroup Mortality Forest Plot.pdf]

Medical Studies Subgroup Mortality Forest Plot

Model	Study name	Odds ratio	Lower limit	Upper limit	p-Value
	Bouman (2002)	1.200	0.480	2.997	0.696
	Payen (2009)	1.522	0.616	3.763	0.363
	Carl (2010)	0.511	0.258	1.010	0.054
	Chon (2012)	0.268	0.079	0.910	0.035
	Jamale (2013)	1.855	0.873	3.939	0.108
	Wald (2015)	0.868	0.381	1.979	0.737
Random		0.904	0.530	1.544	0.712

(Statistics for each study)

Odds ratio and 95% CI

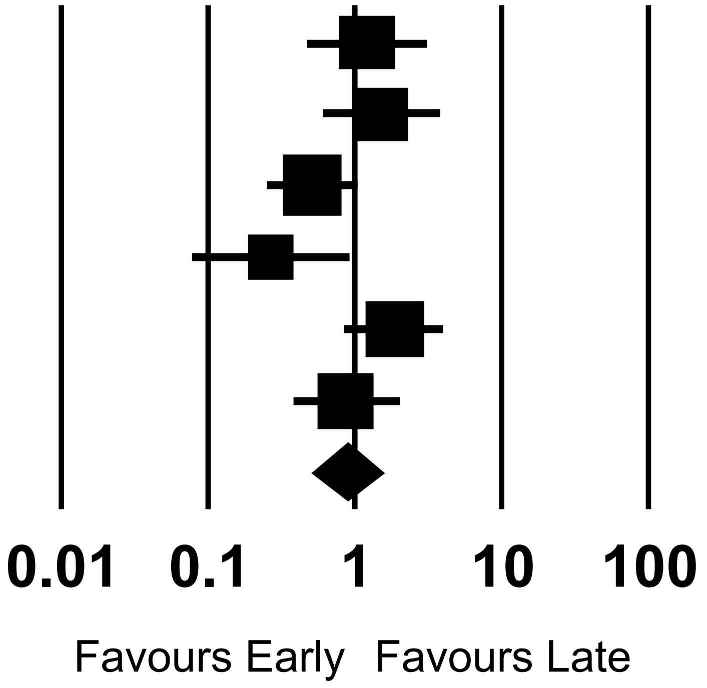

n = 6

Figure 3b. Medical Subgroup Mortality Forest Plot Using Random Effects Model
