# Supplementary material for: The impact of “early” versus “late” initiation of renal replacement therapy in critical care patients with acute kidney injury: a systematic review and evidence synthesis
Source: Crit Care. 2016 May 6;20:122. doi: 10.1186/s13054-016-1291-8 (PMC4858821; doi:10.1186/s13054-016-1291-8)
Supplement: Additional file 5: Figure S4. — a Mortality forest plot of subgroup analysis of high-quality studies based on the definition of “early” according to time criteria (hours or days) (n = 4). b Mortality forest plot of subgroup analysis of high-quality studies based on the definition of “early” according to biochemical parameters (i.e., rising creatinine, uremia, oliguria) (n = 5). (ZIP 121 kb) [file 13054_2016_1291_MOESM5_ESM.zip › Supplementary Figure 4 a and b/Supplementary Index_Figure 4a_Biochemical Subgroup Mortality Forest Plot.pdf]

Biochemical Studies Subgroup Mortality Forest Plot

Model	Study name	Statistics for each study				Odds ratio and 95% CI
		Odds ratio	Lower limit	Upper limit	p-Value	
	Sugahara (2004)	0.028	0.003	0.231	0.001	
	Shiao (2009)	0.260	0.110	0.614	0.002	
	Carl (2010)	0.511	0.258	1.010	0.054	
	Jamale (2013)	1.855	0.873	3.939	0.108	
Random		0.382	0.113	1.288	0.121	

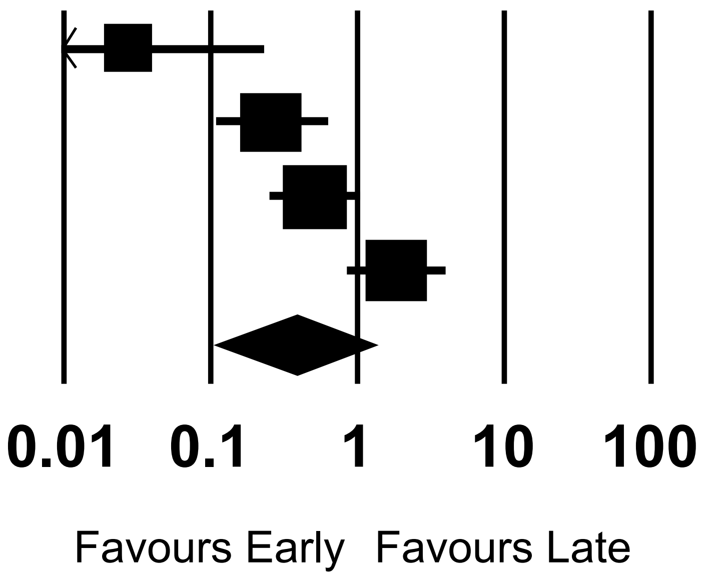

n = 4

Figure 4a. Biochemical Studies Subgroup Mortality Forest Plot Using Random Effects Model
