# Supplementary material for: The impact of “early” versus “late” initiation of renal replacement therapy in critical care patients with acute kidney injury: a systematic review and evidence synthesis
Source: Crit Care. 2016 May 6;20:122. doi: 10.1186/s13054-016-1291-8 (PMC4858821; doi:10.1186/s13054-016-1291-8)
Supplement: Additional file 5: Figure S4. — a Mortality forest plot of subgroup analysis of high-quality studies based on the definition of “early” according to time criteria (hours or days) (n = 4). b Mortality forest plot of subgroup analysis of high-quality studies based on the definition of “early” according to biochemical parameters (i.e., rising creatinine, uremia, oliguria) (n = 5). (ZIP 121 kb) [file 13054_2016_1291_MOESM5_ESM.zip › Supplementary Figure 4 a and b/Supplementary Index_Figure 4b_Time Subgroup Mortality Forest Plot.pdf]

Time-based Studies Subgroup Mortality Forest Plot

Model	Study name	Odds ratio	Lower limit	Upper limit	p-Value
	Bouman (2002)	1.200	0.480	2.997	0.696
	Payen (2009)	1.522	0.616	3.763	0.363
	Chon (2012)	0.268	0.079	0.910	0.035
	Combes (2015)	1.000	0.579	1.727	1.000
	Wald (2015)	0.868	0.381	1.979	0.737
Fixed		0.956	0.670	1.364	0.803
Random		0.937	0.606	1.447	0.768

(Column group "Statistics for each study": Odds ratio, Lower limit, Upper limit, p-Value)

Odds ratio and 95% CI

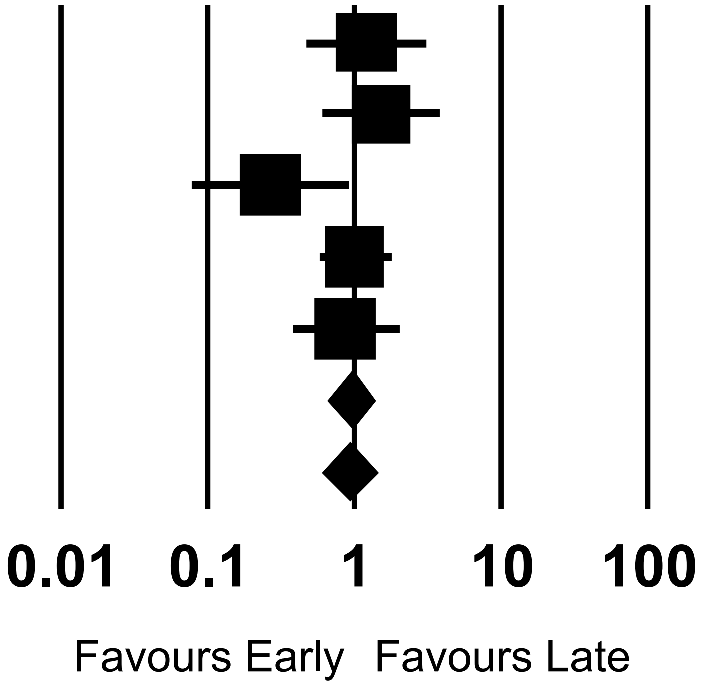

n = 5

Figure 4b. Time-based Studies Subgroup Mortality Forest Plot Using Random Effects Model
